# Design of a high-efficiency brushless wound rotor synchronous machine with reduced torque ripple employing a novel stator winding scheme

**DOI:** 10.1371/journal.pone.0353277

**Published:** 2026-07-17

**Authors:** Muhammad Ahsan ul Haq, Haroon Farooq, Rehan Liaqat, Zafar A. Khan, Abdullah Altamimi

**Affiliations:** 1 Department of Electrical, Electronics & Telecommunication Engineering, University of Engineering and Technology, Lahore (Faisalabad Campus), Punjab, Pakistan; 2 Department of Electrical Engineering (RCET), University of Engineering and Technology, Lahore, Pakistan; 3 Department of Electrical Engineering and Technology, Government College University Faisalabad, Faisalabad, Pakistan; 4 Department of Electrical Engineering, Mirpur University of Science and Technology, Mirpur AJK, Pakistan; 5 Department of Electrical Engineering, College of Engineering, Majmaah University, Al-Majmaah, Saudi Arabia; Sri Venkateswara University College of Engineering, INDIA

## Abstract

This paper presents a novel configuration of a brushless wound rotor synchronous machine (BLWRSM). In the proposed configuration, the stator incorporates a specially designed winding having two series-connected sections with unequal numbers of turns. This unique stator winding (SW) scheme generates an additional subharmonic component (SHC) in the stator magnetomotive force (sMMF), which facilitates brushless excitation of the rotor. The rotor includes a field winding (FW) and a harmonic winding (HW), which are connected through an embedded diode bridge rectifier. The SHC in the sMMF produces an alternating voltage in the HW. This alternating voltage is subsequently rectified to direct current (DC) for exciting FW to realize brushless operation. The proposed configuration, employing a unique SW scheme, is evaluated for an 8-pole, 12-slot BLWRSM using two-dimensional finite element analysis in JMAG Designer. The simulation results validate the proposed concept. In comparison with the existing BLWRSM design, the proposed BLWRSM delivers 4.85% higher output power and 4.9% more average torque while using 50.5% lower input current. Unlike conventional BLWRSM topologies requiring dual inverters, the proposed machine operates with a single three-phase inverter and exhibits 0.69% torque ripples. Due to its brushless and spark-free design, the proposed configuration is well-suited for applications in fire-hazard environments such as aircraft systems and the oil and gas industry.

## 1. Introduction

Permanent magnet synchronous machines (PMSMs) are employed across multiple applications owing to their simple control, robust construction, high efficiency, and the absence of a direct current (DC) supply system to the rotor. However, rising permanent magnet (PM) prices used in rotor construction have increased their cost. [1]. Therefore, attention has been diverted to alternative types of machines, such as hybrid synchronous machines (HSMs) [2,3] and wound rotor synchronous machines (WRSMs). HSMs use less PM material, while the WRSMs use no PMs at all. An HSM combines the features of PMSM and WRSM and employs both PM and electromagnet for torque production in the rotor [4,5]. Due to their low cost, less reliance on rare-earth PM material, and rich control characteristics over a wider speed range, HSMs are a suitable alternative to PMSMs for many applications, like electric vehicles.

Conventional wound rotor synchronous machines (CWRSMs) are excited by an external DC source. Due to this external DC source, these machines have a high power density [6–8] and superior flux-regulation [9,10], making them suitable for EVs. [11]. However, the use of slip rings and carbon brushes introduces maintenance challenges and potential sparking issues, which restrict the suitability of CWRSMs for high-power applications. Moreover, the high field current required for rotor excitation leads to increased power losses, thereby reducing the machine’s overall efficiency. Generally, WRSMs offer several advantages over PMSMs.

1. Removing PMs from the rotor decreases the total cost of the machine.
2. Compared to PMSMs, WRSMs allow enhanced control capability by enabling the adjustment of $I_{d}$, $I_{q}$, and the field current $I_{f}$.
3. The machine is free from PM losses.
4. Enhanced safety is achieved in the machine via direct field control during inverter faults.
5. Demagnetization issues do not exist in WRSMs.

When compared with PMSMs, WRSMs also have some disadvantages, including:

1. Conventional/Brushed WRSMs face an inherent issue with rotor slip rings and brushes. Continuous operation causes the carbon brushes to wear over time, resulting in higher maintenance requirements.
2. Additional system losses result from excitation of the rotor.
3. The rotor experiences copper losses.
4. Adding a field exciter results in higher system costs.

To address the maintenance challenges in CWRSMs arising from the use of carbon-based brushes together with the slip rings, several brushless (BL)WRSM (BLWRSM) configurations were introduced in published studies. Among the various types, one class of solutions incorporates main exciters along with their pilot exciters, particularly in large power rating machines. Although these approaches achieve BL operation, the inclusion of additional components leads to an increase in both the machine’s size and manufacturing cost [12,13]. These limitations have encouraged researchers to explore alternative topologies that either minimize or eliminate the reliance on PMs for BL excitation [6,14,15]. Now, Researchers are currently working to solve the excitation problems of CWRSMs by developing cheaper, simpler, and more effective excitation methods [16–20,21].

In [17], stator windings (SWs) generate a magnetomotive force (MMF) within the air gap comprising both fundamental component (FC) and subharmonic component (SHC). Alternating voltages in the rotor’s harmonic winding (HW) are generated by the SHC of the sMMF. A rotor-mounted rectifier rectifies these voltages to produce a DC supply, which energizes the exciter winding without slip rings and carbon brushes and establishes the rotor’s magnetic field.

In [20], BL operation is realized by employing two SWs: a main SW and an additional SW. The main SW is fed by the FC from Inverter 1, whereas the additional SW is energized with a third-harmonic current from Inverter 2. However, employing dual inverters along with dual SWs raises the overall system cost. In addition, the copper losses associated with two SWs, together with the power losses in both inverters, lead to a reduction in the overall machine efficiency.

**Fig 1:**
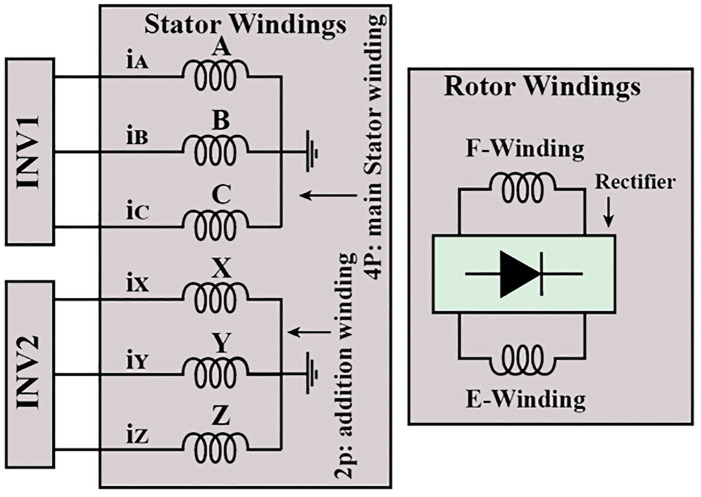
Dual stator winding and two-inverter configuration designed for BL operation of WRSM.

A BLWRSM based on subharmonic excitation is presented in [22], utilizing multi-pole armature and rotor windings to achieve BL field excitation via a rotating rectifier. As illustrated in Fig 1, the system requires two separate inverters. The topology is validated using 2D FEM and compared with an existing dual three-phase design in terms of torque, torque ripple, induced current, and efficiency.

A 48-pole, 36-slot BLWRSM with concentrated SWs is reported in [23]. In this design, the SHC present in the sMMF creates alternating voltage within HW, whereas the FC available in sMMF works together with the rotor’s main magnetic field to generate electromagnetic torque. BL operation is facilitated by incorporating an additional rotor’s excitation winding. To deliver the necessary DC for field winding (FW), a rectifier is installed between the additional rotor excitation winding and the main FW.

Authors of [24] introduced a single-inverter-based BL topology in which the FC and the SHC are generated in the sMMF. The topology has HWs on both the stator and rotor. On the stator side, six switches are utilized to produce the SHC in the sMMF. As a result, AC voltages are induced in the rotor HWs. Following rectification, this AC voltage provides brushless DC excitation to the rotor FW.

Authors of [25] presented a BL self-excitation topology for the WRSM. Their design employs a multiphase stator to generate two rotating magnetic fields. These rotating magnetic fields correspond to specific harmonic orders, enabling magnetic interaction with the rotor’s multiple windings. The rotor assembly incorporates transformer windings, FW, and a rectifier. The rectifier rectifies the produced alternating currents (ACs) within the transformer windings to energize the FW with DC.

Reference [26] discusses a dual-mode WRSM for adjustable-speed applications, integrating the benefits of both conventional and BLWRSM. The machine provides constant torque and constant power in Mode-I and Mode-II respectively. The mode transition is controlled through a thyristor-based circuit. Analytical derivation of air-gap MMF, finite element analysis (FEA), and experiments were performed on a 1-hp model to validate the proposed concept.

A large number of BL topologies for WRSMs have been discussed in the literature by various researchers. These topologies differ in terms of winding configuration, inverter requirements, and excitation methods. A comparative summary of recently reported BLWRSM topologies, along with the proposed topology, is presented in Table 1.

**Table 1:** Comparative summary of recent BLWRSM topologies and the proposed topology.

Reference	Year	Topology	Limitations/Remarks
[27]	2016	The topology employs two inverters and permanent magnets.	Two inverters are required in the proposed topology, which increases the system’s dimensions and leads to higher costs.
[28]	2020	An inverter with two extra diodes generates fundamental and third-harmonic MMF.	An inverter with two extra diodes increases the cost.
[24]	2020	BLWRSM employs a stator HW.	The use of two HWs (on both stator and rotor) increases structural complexity, while the requirement of six stator-side switches for SHC generation further adds to system complexity and cost.
[16]	2020	A hysteresis controller is used to generate the third-harmonic in the stator MMF (sMMF) alongside the fundamental MMF.	Hysteresis controller to generate the third-harmonic adds control complexity and increase switching losses, making the system less efficient.
[29]	2021	BL operation of WFSM by inducing a third-harmonic component.	Two inverters operating at different frequencies to supply the fundamental and third-harmonic components increase the control and hardware requirements of the system. Consequently, the overall system becomes more complex and costly.
[30]	2022	The proposed machine achieves BL operation using a SHC. It has two divided SWs powered by two inverters, and five rotor switches to change modes.	Two inverters and five rotor switches increase system complexity and cost, making the machine more difficult to control and manufacture.
[31]	2022	Hysteresis controller generates the third-harmonic in the sMMF.	Additional controller adds cost, and in hybrid mode, the core exceeds 2.14 T saturation.
[32]	2023	BL operation of a wound rotor vernier motor is achieved using an additional single-phase auxiliary winding alongside the three-phase SW.	Additional single-phase auxiliary winding increases the machine’s complexity and manufacturing cost. It also requires extra control circuitry, which can complicate the overall system design.
[23]	2023	Subharmonic is used to achieve BL operation of the machine. The proposed design employs an outer-rotor structure.	Outer-rotor configuration increases the overall size of the machine. Additionally, the machine produces high torque ripples during spin mode.
[22]	2023	BLWRSM based on subharmonic excitation	Two separate inverters and two SWs increase the cost.
[33]	2023	The subharmonic based topology uses two stators in its design.	Two stators increase the overall size and cost.
[34]	2025	BL operation is achieved using the third-harmonic component.	High torque ripples and low efficiency are drawbacks in this design. Moreover, the core experiences more heating due to magnetic flux density reaching 2.1887 T.
Proposed design	2025	Two series-connected sets of unequal turns in SW generate an additional SHC in the sMMF, enabling BL operation.	Neither a dual inverter nor a double-layer SW is required in the proposed design. It does not require any controller, single-phase additional SW, additional stator HW, or a double stator. The machine contains only a single-layer winding, which is supplied either by a conventional AC source or by a single inverter.

The literature review indicates that most existing BL topologies primarily depend on dual inverters or additional windings, which increase the machine’s price. To fill this gap, this paper presents the following contributions for a novel BLWRSM.

- It utilizes a unique SW configuration with two series-connected winding sets having unequal numbers of turns. This SW configuration helps generate an additional SHC in the sMMF.
- It achieves the BL functioning of WRSM utilizing SHC in the sMMF.
- The SW of the proposed machines requires a single 3-phase inverter for its operation, thereby eliminating the need for a dual inverter, which reduces both system cost and size.
- Its rotor arrangement contains HW and FW. The SHC links with HW, inducing AC voltages within it. A rotating rectifier is employed to convert these voltages into DC, which is injected into the FW on the rotor to establish the machine’s main magnetic field.
- The proposed configuration exhibits more average torque with less SW current in comparison to the existing BLWRSM design [26].
- The proposed topology achieves significantly lower torque ripples in comparison to the existing configuration [26].

The proposed configuration is validated using 2-dimensional (2-D) FEA on an 8-pole 12-slot motor. The manuscript is arranged as follows in the subsequent sections.:

- The topology, motor design, and operating principle of the proposed BL configuration are outlined in Section II.
- Section III presents the 2-Dimensional finite element (2-D FEA) of the suggested machine in no-load and load conditions.
- Concluding remarks are stated in Section IV.

## 2. Proposed BL machine topology, machine design, and principle of operation

### 2.1. Proposed BL machine topology

**Fig 2:**
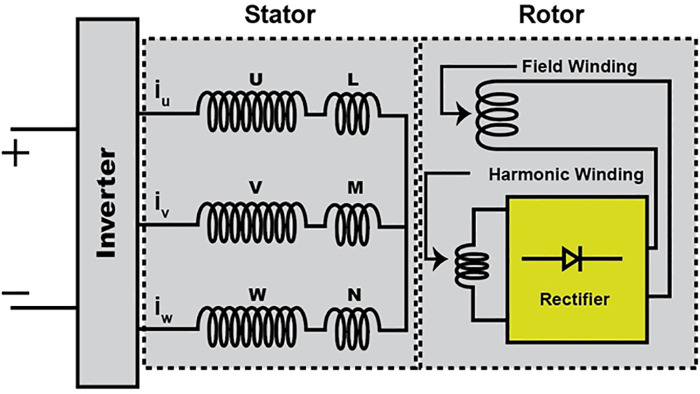
Schematic representation of proposed BL topology.

The proposed BL machine employs a 3-phase single-layer SW, which is composed of two sets: UVW and LMN. The two sets are connected in cascade in such a way that the turn count in the UVW set is twice that of the LMN set. The proposed topology is presented in Fig 2. The 3-phase current in the balanced condition is given to the SW of the suggested machine. Because of the unique single-layer SW configuration of the proposed topology, two dominant components of sMMF are produced. These are SHC and FC. The SHC is spent to give DC to the rotor’s FW.

Two windings (FW and HW) are present on the rotor. The FW is designed with 8 poles, while the HW has 4 poles. The two windings are interconnected by means of a rotating bridge rectifier, which facilitates AC-to-DC conversion between them. Within the HW, AC voltages are generated by the SHC of the sMMF. These voltages are converted into DC by the rotating bridge rectifier, which serves as the excitation input for the rotor FW. The SW winding is arranged in an 8-pole configuration, and to ensure effective torque production, both the FW and the SW have the same number of poles.

### 2.2. Machine design

**Fig 3:**
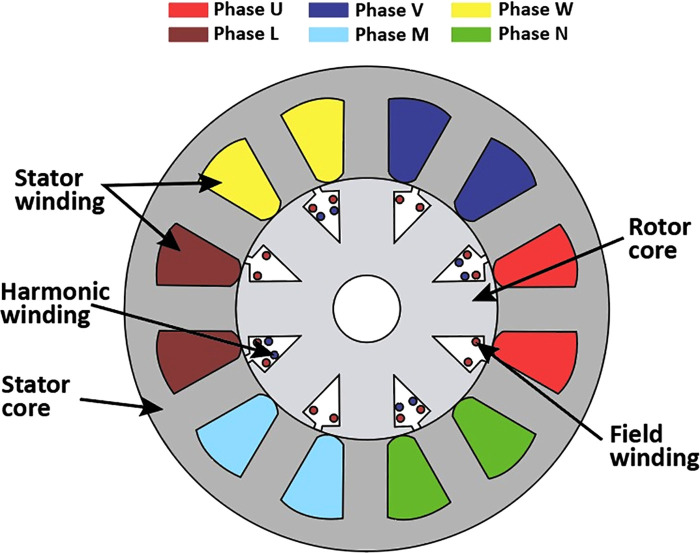
Layout of stator and rotor windings in the proposed BLWRSM.

A machine with 8 poles and 12 stator slots, incorporating a single-layer SW, is utilized to validate the proposed BL topology. The configuration of the proposed BL machine, showing the rotor winding and SW layout, is presented in Fig 3. A single 3-phase inverter is used to supply a balanced current to the SW of the proposed BLWRSM. For realizing the BL function of the proposed topology, the SW is arranged so that the (U, L), (V, M), and (W, N) pairs are located in front of each other and are connected in cascade with each other, as shown in Fig 3. Table 2 describes the specifications of the proposed machine.

**Table 2:** Machine design specifications.

Parameter	Units	Proposed BLWRSM
Power rating	W	746
Speed of the machine	rev/min	900
Outer diameter (stator core)	cm	17.7
Inner diameter of the stator core	cm	9.5
Air gap	mm	0.5
Diameter of the proposed machine’s shaft	cm	2.5
Length of the core stack	cm	8
Poles of the SWs	–	8
Poles of FW	–	8
Slots for SW	–	12
Per phase turns in UVW set of SW	–	160
Per phase turns in LMN set of SW	–	80
Turns for FW	–	224
Poles in HW		4
Turns for HW	–	32
Conductor material for winding		Copper
Material for core	–	50H1300

In this manuscript, instead of using a second inverter or any external controller with a single inverter to generate the SHC, a special type of SW is employed that inherently produces SHC along with the FC. The proposed SW is designed such that each phase consists of two series-connected coils. One coil set (U-coil set) has double the number of turns compared to the other coil set (L-coil set). Similarly, all three phases are constructed using the same configuration. This unique configuration helps produce the fundamental MMF along with a strong SHC.

**Fig 4:**
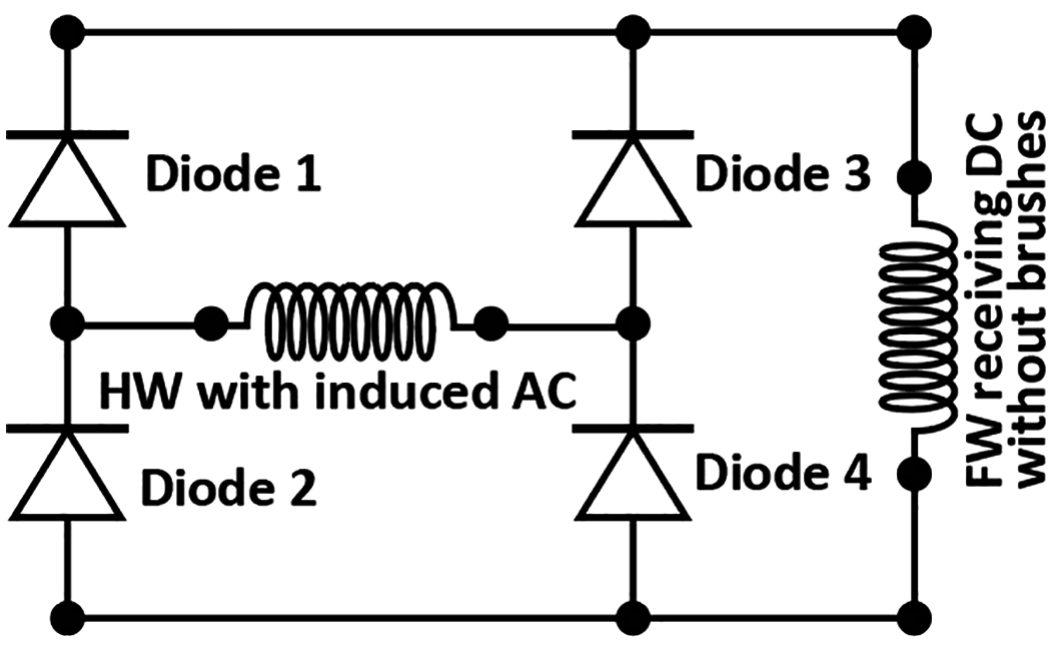
Rotor schematic of the proposed BLWRSM.

**Fig 5:**
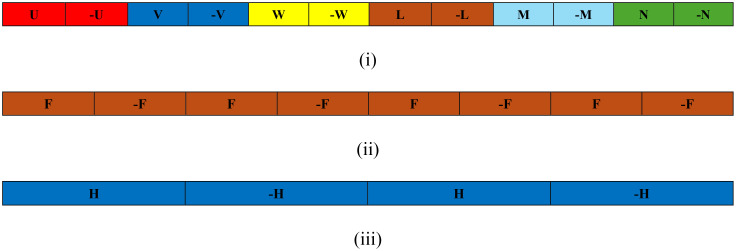
(i) Configuration of SW (ii). Configuration of FW (iii). Configuration of HW.

The rotor schematic of the proposed machine is shown in Fig 4. The rotor contains HW and FW in such a way that the pole pitch of FW is halved compared to the pole pitch of HW. Hence, the HW is designed with 4 poles, while the FW has 8 poles. An AC voltage is generated in HW by the SHC of air gap sMMF. A bridge rectifier is employed to convert the AC voltage into DC. The rectified DC is injected into the main rotor FW. Figs 5(i)–5(iii) present the configurations of the SW, rotor FW, and rotor HW of the proposed machine, respectively.

### 2.3. Operating principle

**Fig 6:**
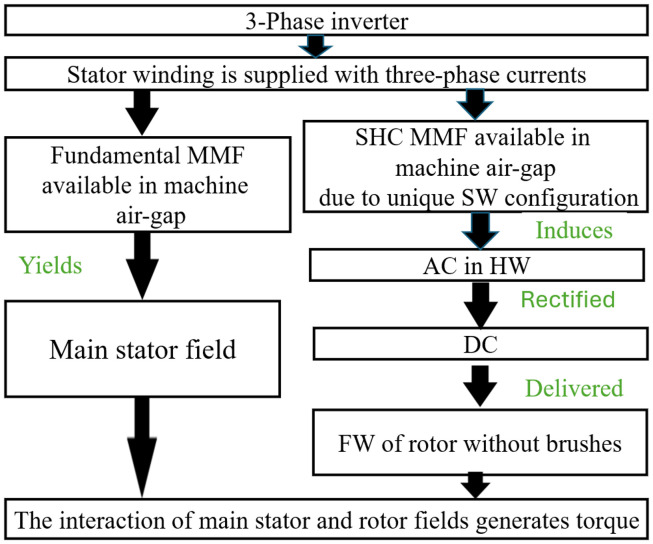
Principle of operation of the proposed configuration.

The principle of operation of the proposed BL machine is illustrated in the block diagram shown in Fig 6. A single inverter is sufficient to operate the machine, with no additional inverter required. From the sMMF, the available SHC enables BL operation of the proposed machine. Both (FC & SHC) of the sMMF are produced by a 3-phase single-layer SW divided into two series-connected winding sets. These windings have unequal numbers of turns. The winding UVW has twice the turns of winding LMN, and is powered by an inverter. The SHC generates AC voltages in HW, which are then fed to a rectifier that converts them into DC to energize the machine’s FW, thereby establishing the main rotor field.

**Fig 7:**
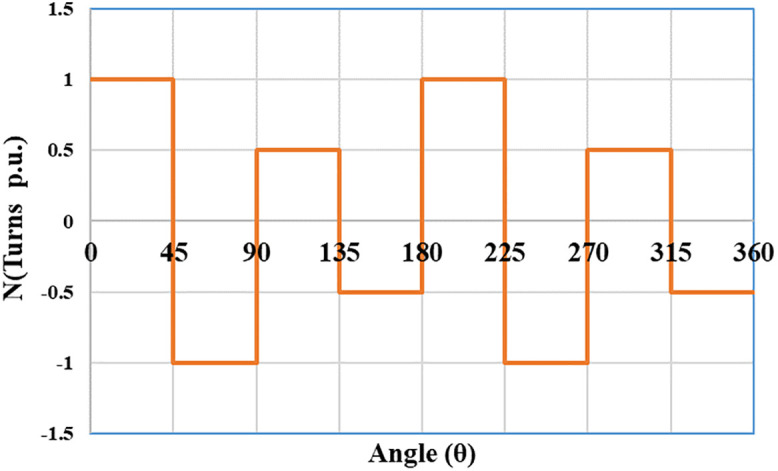
Phase U winding function.

The air-gap MMF generation is analytically investigated for an 8-pole configuration employing concentrated full-pitch SWs. The rectangular winding function corresponding to phase U is illustrated in Fig 7. This distribution can be mathematically represented by a Fourier series expansion consisting solely of odd harmonic components.

The SW is energized with 3-phase currents, expressed as:


$$\begin{array}{c} i_{u}(t)=I_{u,max}\;sin\omega_{e}t \end{array}$$ (1)

$$\begin{array}{c} i_{v}(t)=I_{v,max}\;sin(\omega_{e}t-\frac{\text{2}\pi }{\text{3}}) \end{array}$$ (2)

$$\begin{array}{c} i_{w}(t)=I_{w,max}\;sin(\omega_{e}t+\frac{\text{2}\pi }{\text{3}}) \end{array}$$ (3)

where $I_{u,max}$ $I_{v,max}$ and $I_{w,max}$ represents the peak values of the currents supplied by the inverter in the respective phases and $I_{u,max}=I_{v,max}=$ $I_{w,max}=I_{max}$. The acronyms t and $\omega_{e}$ denote the time and angular frequency, respectively.

The rotating MMF for m phase system can be expressed as:


$$\begin{array}{c} F\;(\theta ,j)=\sum_{j=\text{1}}^{m}N_{j}(\theta ).\;i_{j}(t) \end{array}$$ (4)

Here $N_{j}(\theta )$ is the winding function for the phase j, which depends on the angular measure (θ) around the machine’s air gap.

For the 3-phase system, the MMF expression can be represented as:


$$\begin{array}{c} F_{UVW}(\theta ,i)=\left\{N_{U}(\theta ).i_{U}(t)\right\}+\left\{N_{V}(\theta ).i_{V}(t)\right\}+\left\{N_{W}(\theta ).i_{W}(t)\right\} \end{array}$$ (5)

The winding functions for phases U, V, and W, can be presented as:


$$\begin{array}{c} N_{U}(\theta )=\frac{\text{2}N}{\pi }\left\{sin\theta +\frac{\text{1}}{\text{3}}sin\text{3}\theta \right\}+\frac{N}{\pi }\left\{cos\left(\frac{\theta }{\text{2}}\right)+sin\theta \right\} \end{array}$$

$$\begin{array}{c} N_{V}(\theta )=\frac{\text{2}N}{\pi }\left\{sin\left(\theta -\frac{\text{2}\pi }{\text{3}}\right)+\frac{\text{1}}{\text{3}}sin\text{3}\theta \right\}+\frac{N}{\pi }\left\{cos\left(\frac{\theta -\frac{\text{2}\pi }{\text{3}}}{\text{2}}\right)+\text{sin}\left(\theta -\frac{\text{2}\pi }{\text{3}}\right)\right\} \end{array}$$

$$\begin{array}{c} N_{W}(\theta )=\frac{\text{2}N}{\pi }\left\{sin\left(\theta +\frac{\text{2}\pi }{\text{3}}\right)+\frac{\text{1}}{\text{3}}sin\text{3}\theta \right\}+\frac{N}{\pi }\left\{cos\left(\frac{\theta +\frac{\text{2}\pi }{\text{3}}}{\text{2}}\right)+\text{sin}\left(\theta +\frac{\text{2}\pi }{\text{3}}\right)\right\}\; \end{array}$$ (6)

From expressions (1), (2), (3), and (6), expression (5) for MMF can be represented as:


$$\begin{array}{cc} F_{UVW}(\theta ,i)=\; & \frac{\text{3}}{\pi }NI_{max}\left[\text{sin}\theta .\;sin\omega t+sin\left(\theta -\frac{\text{2}\pi }{\text{3}}\right).sin\left(\omega t-\frac{\text{2}\pi }{\text{3}}\right)+\;sin\left(\theta +\frac{\text{2}\pi }{\text{3}}\right).sin\left(\omega t+\frac{\text{2}\pi }{\text{3}}\right)\right]\\ & \;\;+\frac{\text{1}}{\pi }NI_{max}\left[\text{cos}\left(\frac{\theta }{\text{2}}\right).sin\omega t+cos\left(\frac{\theta -\frac{\text{2}\pi }{\text{3}}}{\text{2}}\right).sin\left(\omega t-\frac{\text{2}\pi }{\text{3}}\right)+cos\left(\frac{\theta +\frac{\text{2}\pi }{\text{3}}}{\text{2}}\right).sin\left(\omega t+\frac{\text{2}\pi }{\text{3}}\right)\right] \end{array}$$ (7)

The second term in expression (7) corresponds to the SHC of the sMMF, which facilitates the rotor’s BL excitation. As the SHC plays a crucial role in achieving BL excitation, tight tolerances are recommended during the machine manufacturing to avoid increased torque ripple, vibration, and efficiency reduction.

The sMMF of the proposed BL design has dominant 1- and 0.5-order harmonics, with the 0.5-order harmonic inducing AC voltages in the rotor HW. For an 8-pole machine operating at 900 rpm with a 60 Hz supply, the angular frequency of the FC ($\omega_{FC}$) is calculated as


$$\begin{array}{c} \omega_{FC(at\;\text{6}\text{0}\;Hz)}=\frac{\text{2}\pi }{\text{60}}\times \text{900 }radian/second=\text{94}.\text{25 }radian/second \end{array}$$ (8)

Similarly, the SHC’s angular frequency, denoted as $\omega_{SHC}$ is determined as


$$\begin{array}{c} \omega_{SHC(\text{3}\text{0}\;Hz)}=\frac{\omega_{FC}}{sh}=\frac{\text{94}.\text{25}}{\text{0}.\text{5}}=\text{188}.\text{5}\;radian/second \end{array}$$ (9)

Where the subharmonic number sh represents the subharmonic order, and the angular frequency of the fundamental MMF is half the SHC’s angular frequency. From these equations, it is concluded that the SHC rotates at $\omega_{SHC(\text{3}\text{0}\;Hz)}$ which is two times the $\omega_{FC(at\;\text{6}\text{0}\;Hz)}$. This variation in speed induces AC voltages in HW, which are then rectified and provided to the rotor’s FW without slip rings and brushes to realize BL operation.

**Fig 8:**
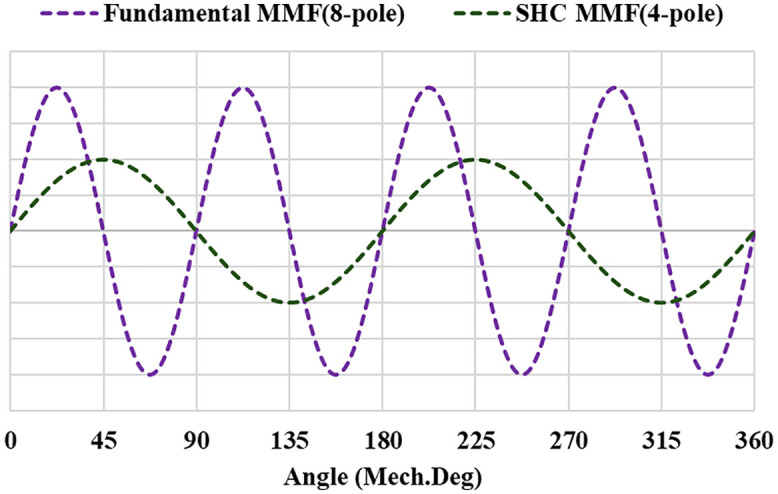
Fundamental and SHC of the stator MMF.

Fig 8 depicts the waveforms of the SHC and FC over a complete mechanical cycle of 360°. The HW has the same number of poles as the SHC, while the FW poles correspond to the FC poles. Specifically, the SHC shows four poles, while the FC displays eight poles per cycle, in accordance with the design parameters presented in Table II for the proposed machine. Additionally, the frequency of the FC is twice that of the SHC, with both the FW and FC showing eight poles, and both the HW and SHC showing four poles in the proposed configuration.

## 3. performance assessment by 2-D FEA

### 3.1. Proposed blwrsm no-load analysis

**Fig 9:**
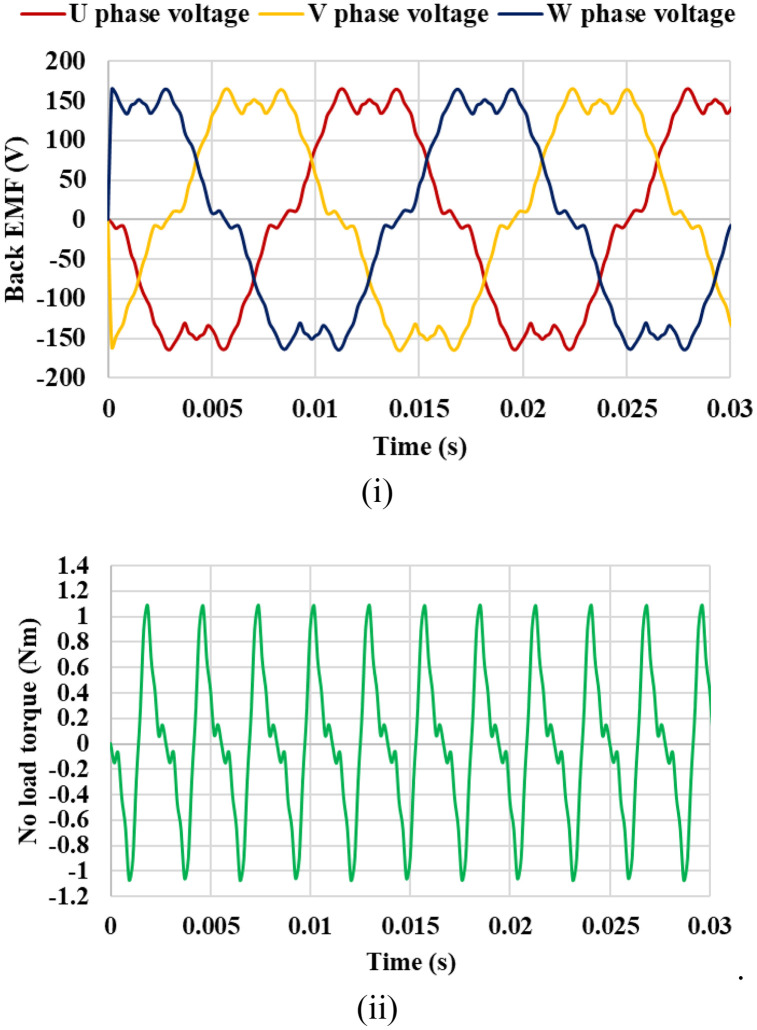
(i) Back-EMF of the proposed BLWRSM, (ii) Proposed BLWRSM no-load torque.

An analysis under no-load conditions was conducted to evaluate the no-load torque and back-EMF of the proposed configuration. To achieve this, the rotor FW was energized with a DC field current of 1 A, and the motor was run at synchronous speed (900 rpm). The corresponding back-EMF and no-load torque waveforms are depicted in Figs 9(i) and 9(ii), respectively. The proposed machine’s no-load torque was −0.00036 Nm. The back-EMF and no-load torque results are presented in Table 3.

**Table 3:** Results of non-load analysis for the proposed BL configuration.

Attribute	Units	Proposed BLWRSM
Back EMF	V_rms_	113.87
No-load torque	Nm	−0.000360

### 3.2. Proposed BLWRSM load analysis

**Fig 10:**
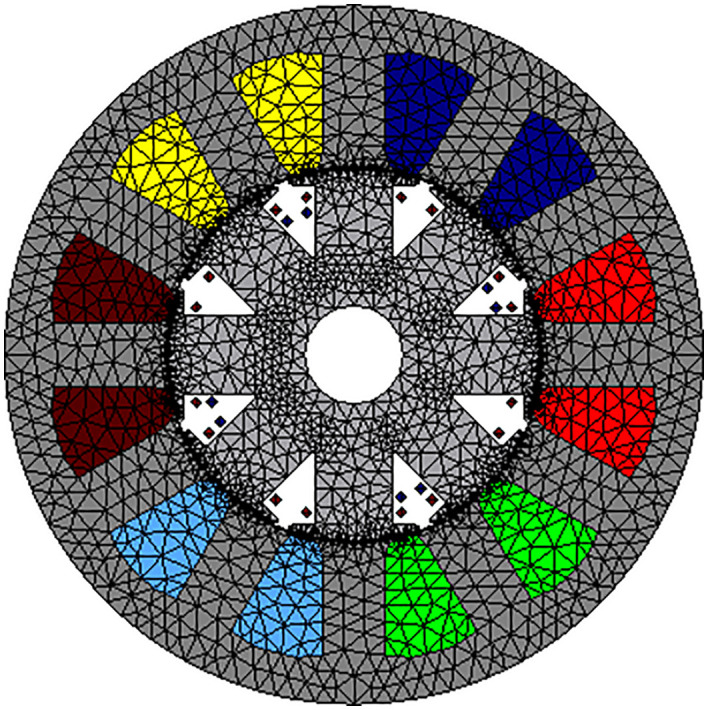
Finite element mesh applied to the proposed BLWRSM for performing load analysis.

**Fig 11:**
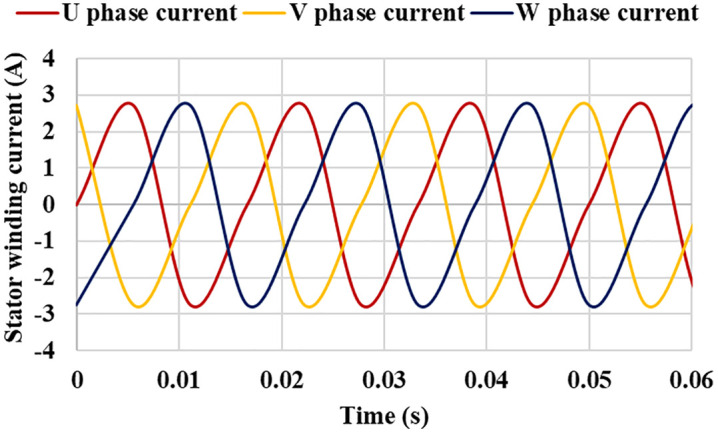
3-phase stator current supplied to the proposed BLWRSM under load conditions.

**Fig 12:**
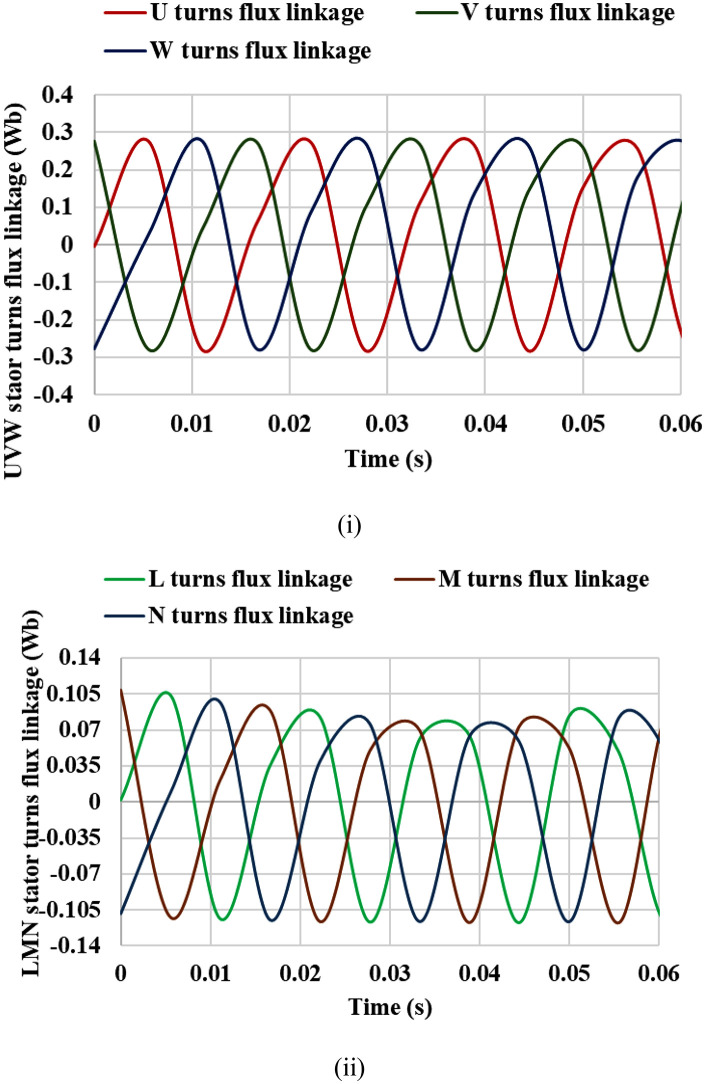
(i) Flux linkage with the UVW stator turns in the proposed configuration (ii) Flux linkage with the LMN stator turns in the proposed configuration.

To validate and investigate the proposed BL topology, 2-D FEA was conducted using JMAG Designer. The meshing employed in the BLWRSM is illustrated in Fig 10. The RMS per-phase current supplied to SW is 2.227 A, and currents applied to the three-phase SW are shown in Fig 11. The machine operates at a synchronous speed of 900 rpm. In the proposed configuration, two dominant components are present in the sMMF. These are SHC and FC. The main stator magnetic field of the proposed machine is produced by the FC, whereas AC voltages are developed in HW by the SHC of the air-gap sMMF. Therefore, the SHC must have sufficient and reliable magnitude to effectively generate the AC voltages in the HW of the rotor. These generated AC voltages are converted to supply DC for FW excitation, which in turn establishes the primary rotor field. The flux linkages of the SWs in the proposed BL configuration are presented in Figs 12(i)–(ii).

**Fig 13:**
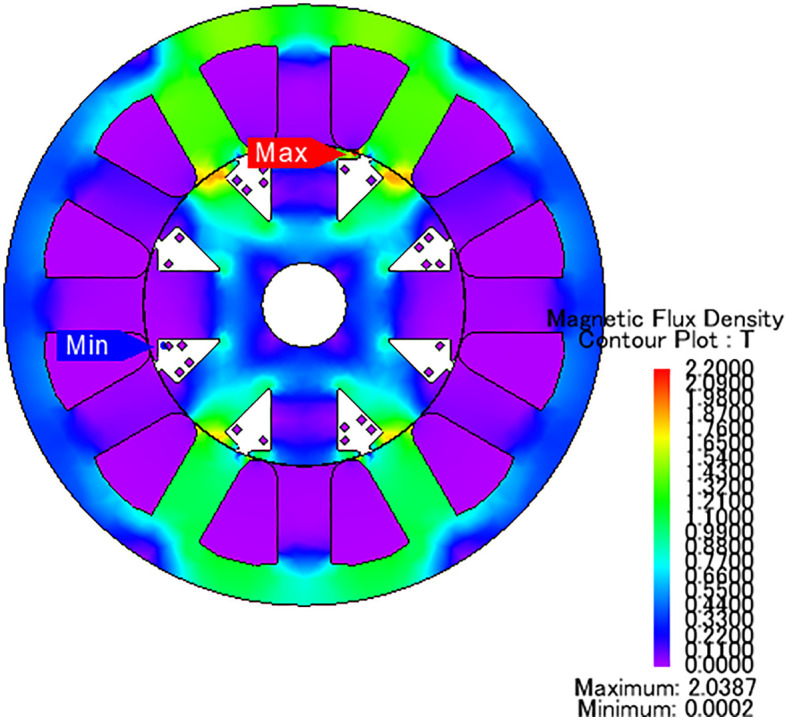
Proposed BLWRSM Flux density distribution under loaded operation.

**Fig 14:**
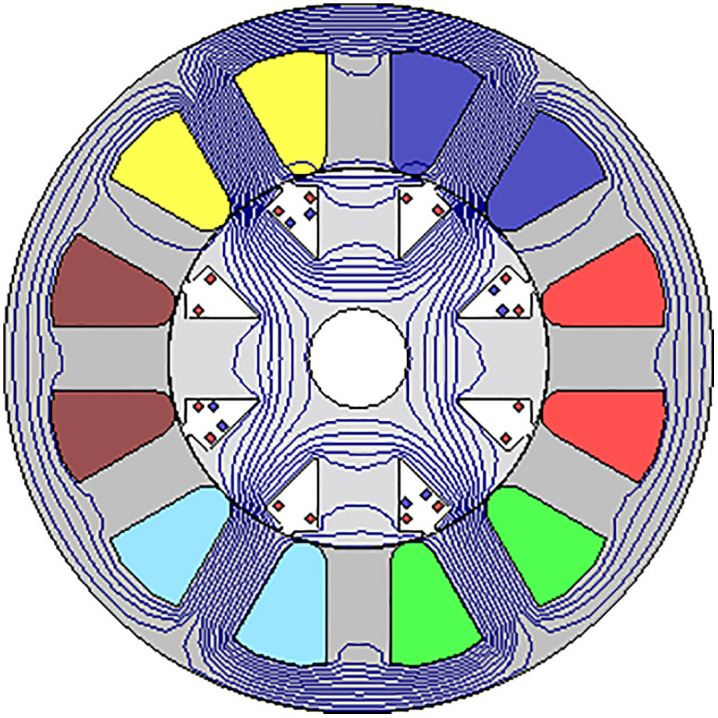
Flux distribution of the proposed BLWRSM during loaded operation.

The flux density of the proposed configuration is presented in Fig 13. The flux density distribution helps identify the saturated regions within the core. Similarly, under loaded conditions, the flux lines of the proposed BLWRSM are shown in Fig 14. The magnetic lines form closed loops, passing from the stator through the rotor and returning, thereby indicating a complete magnetic circuit.

**Fig 15:**
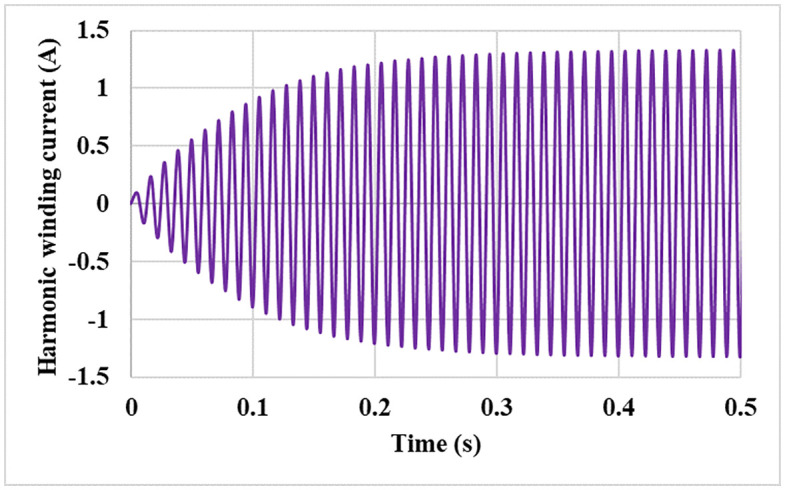
Proposed BLWRSM harmonic winding current profile.

**Fig 16:**
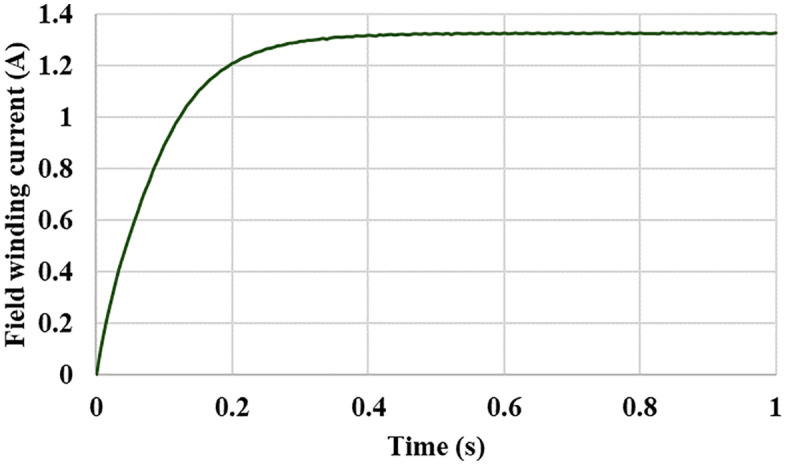
Proposed BLWRSM field winding current profile.

In the proposed motor, an AC voltage is generated in the HW by the 30 Hz SHC and subsequently converted to DC via the rotor-mounted rectifier to supply DC to FW, thereby removing the need for an external DC source. Therefore, the proposed BL configuration operates without slip rings and carbon brushes. At t = 0.3 s, both the rotor-developed torque and FW current reach steady-state conditions. Fig 15 illustrates the AC waveform in the HW for the proposed BLWRSM design, whereas the DC currents in the FWs are presented in Fig 16.

For the proposed motor, the RMS AC induced in the rotor HW is 1.3232 A, while the DC supplied from the rectifier to the rotor FW is 1.323 A. Compared with existing topologies [22,23,35–37], the HW and the rotor FW currents in the proposed configuration are significantly lower. A comparison of the percentage reduction in AC in rotor HW and DC in rotor FW in the proposed design with respect to (w.r.t.) existing designs is presented in Table 4. Lower current levels directly reduce the conduction losses and heat generation in the rotating diode bridge rectifier, which contributes to improved thermal performance and enhanced reliability during prolonged operation. Accordingly, the proposed topology is expected to maintain safe thermal performance under full-load conditions.

**Table 4:** Reduction in harmonic and field winding currents in the proposed toplolgy with respect to existing works.

Topology	AC in rotor harmonic winding (A_RMS_)	DC in rotor field winding (A)	AC reduction in proposed design w.r.t. existing designs (%)	DC reduction in proposed design w.r.t. existing designs (%)
Proposed	1.3232	1.323	—	—
[23]	5.7 A	6.26 A	−76.79%	−78.87%
[22]	8.32	11.94	−84.1%	−88.92%
[35]	5.3	9.2	−75.03%	−85.62%
[36]	22.62	24.55	−94.15%	−94.61%
[37]	5.77	10.29	−77.07%	−87.14%

**Fig 17:**
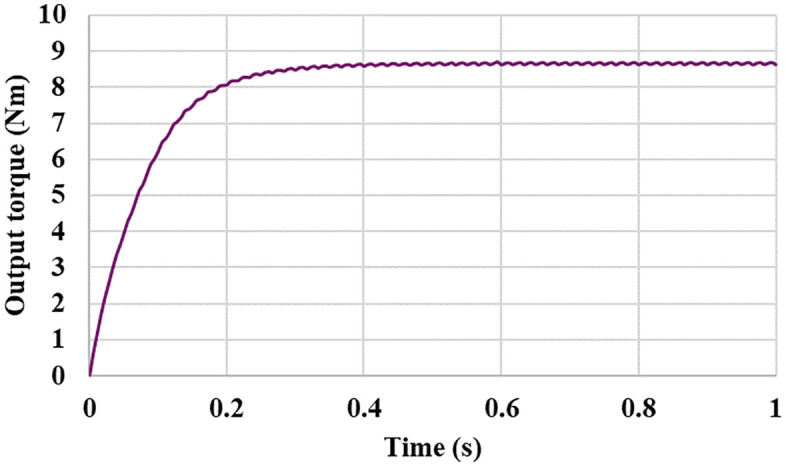
Proposed BLWRSM output torque.

**Fig 18:**
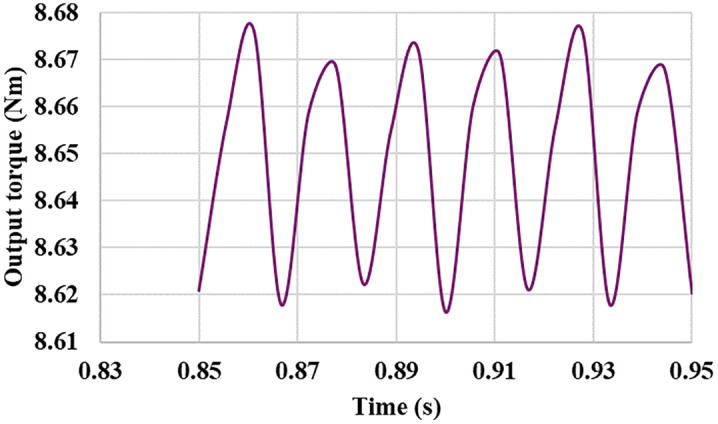
Magnified view of the proposed BLWRSM output torque.


Fig 17 illustrates the torque profile of the proposed BLWRSM. The machine provides an average torque of 8.644 Nm with a torque ripple of 0.694%. The minimal torque ripple demonstrates the high-performance capability of the proposed design. Fig 18 provides a zoomed view of the output torque in the steady state. Consistent with the BLWRSM topologies discussed in [31,38], torque ripples are observed in the proposed motor and are quantified using (10).

$$\begin{array}{c} \%T_{rip}=\left[\frac{T_{max}-T_{min}}{T_{avg}}\right]\times \text{1}\text{0}\text{0} \end{array}$$ (10)

Here, $T_{\text{max}}$ is the maximum torque, $T_{\text{avg}}$ is the average torque, and $T_{\text{min}}$ is the minimum torque produced by the proposed machine, while torque ripple is expressed as a percentage, denoted by $\%T_{rip}$.

Using (10), the torque ripples of the proposed machine are.


$$\begin{array}{c} \%T_{rip.}=\left[\frac{\text{8}.\text{6}\text{8}-\text{8}.\text{6}\text{2}}{\text{8}.\text{6}\text{4}\text{4}}\right]\times \text{1}\text{0}\text{0} \end{array}$$

$$\begin{array}{c} \%T_{ripples}=\text{0}.\text{6}\text{9}\text{4}\% \end{array}$$

### 3.3. Comparative analysis of the proposed BL configuration with the CWRSM

A comparison between the proposed BLWRSM and a conventional WRSM (CWRSM) was conducted to evaluate the advantages of the suggested BL topology. The CWRSM is equipped with brushes and an external DC supply, and it serves as a benchmark for assessing the performance of novel BLWRSM configurations. Several studies, such as [7], have compared their proposed design against CWRSM. A BLWRSM that achieves a comparable average torque to a CWRSM with reduced torque ripple is generally regarded as a superior design.

To ensure a fair comparison, the following design constraints were implemented on both machines:

- The same 50H1300 core material was used in both machines.
- The stator inner as well as outer diameters were identical for both machines.
- The air-gap length was set to 0.5 mm.
- The shaft diameter was 2.5 cm.
- Both machines were run at 900 rev/min.
- The number of FW turns and SW turns per phase was maintained the same.
- Both designs employed a stack length of 8 cm.

**Fig 19:**
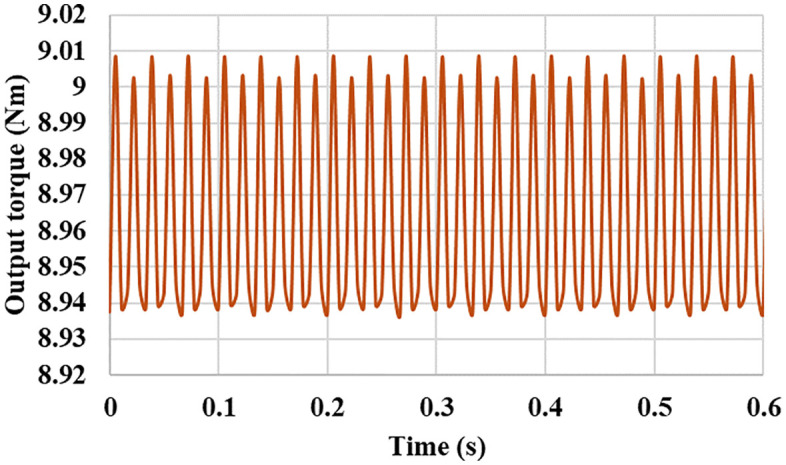
Output torque of CWRSM.

**Fig 20:**
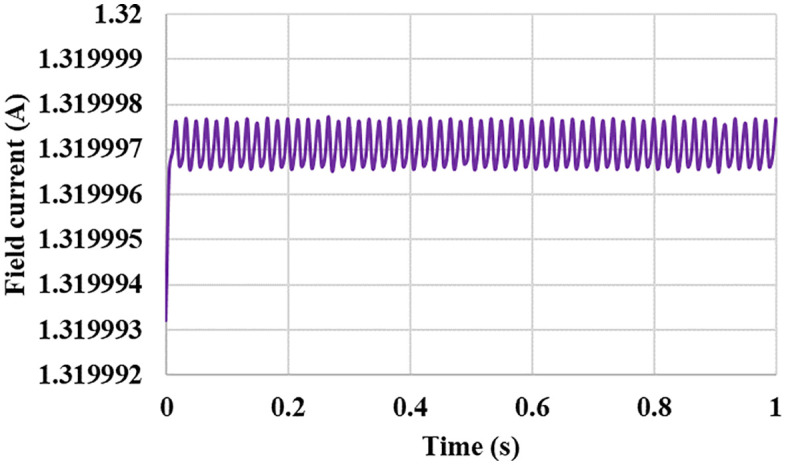
Field current of CWRSM.

For the CWRSM, there are equal turns in UVW and LMN windings. However, rotor FW receives excitation from an external DC supply using slip rings and carbon brushes. There is no need for brushes or slip rings in the proposed BL topology. The proposed BLWRSM topology employs HW on the rotor to induce AC voltages in it. From Fig 19, the output torque of CWRSM can be viewed. Fig 20 shows the current given to the rotor FW of CWRSM directly from an external DC supply using brushes.

Table V presents the comparison between the proposed BLWRSM and CWRSM. Equations (11)–(15) can be used to evaluate the efficiency (%η) of the machine.


$$\begin{array}{c} \%\eta =\left[\frac{P_{out}}{P_{in}}\right]\times \text{1}\text{0}\text{0} \end{array}$$ (11)

$$\begin{array}{c} \%\eta =\left[\frac{P_{out}}{P_{out}+P_{loss}}\right]\times \text{1}\text{0}\text{0} \end{array}$$ (12)

Where


$$\begin{array}{c} P_{out}=\left[\frac{\text{2}\pi }{\text{6}\text{0}}\right]\times N\tau \end{array}$$ (13)

$$\begin{array}{c} P_{loss}=P_{cor.}+P_{cop.} \end{array}$$ (14)

$$\begin{array}{c} \text{and }P_{cop.}=P_{s,cop.}+P_{f,cop.}+P_{h,cop.} \end{array}$$ (15)

Here $P_{in}$, $P_{loss}$ and $P_{out}$ represent the input power, power loss, and output power of the proposed machine, respectively. Similarly, τ and N denote the output torque (Nm) and motor speed (rev/min), respectively. Two types of losses occur in the proposed machine: core losses, denoted as $P_{\text{cor}}$, and copper losses, denoted as $P_{\text{cop}}.$The copper loss occurs in FW, SW, and HW, which are respectively represented by $P_{f,cop.},P_{s,cop.\;}$ and $P_{h,cop.}$. Using equations (11)–(15), %η can be computed as:


$$\begin{array}{c} \%\eta =\left[\frac{\left\{\left(\frac{\text{2}\pi }{\text{6}\text{0}}\right)\times \;\text{9}\text{0}\text{0}\times \text{8}.\text{6}\text{4}\text{4}\right\}}{\left\{\left(\frac{\text{2}\pi }{\text{6}\text{0}}\right)\times \;\text{9}\text{0}\text{0}\times \text{8}.\text{6}\text{4}\text{4}\right\}+\left\{\text{5}\text{6}+\text{3}\text{5}.\text{7}\text{1}+\text{0}.\text{5}\text{6}+\text{3}.\text{9}\text{2}\text{1}\right\}}\right]\times \text{1}\text{0}\text{0}=\text{8}\text{9}.\text{4}\text{4}\% \end{array}$$

The results summarized in Table 5 show that the proposed BLWRSM achieves comparable performance with respect to conventional WRSM. The average torque of the CWRSM is 8.963 Nm, whereas the proposed BLWRSM produces 8.644 Nm. In terms of efficiency, the CWRSM achieves 89.988%, while the proposed BLWRSM reaches 89.44%. This corresponds to a very small efficiency reduction of approximately 0.61% in the proposed BL topology. The slight reduction is mainly attributed to additional copper losses associated with the HW used for BL excitation in the proposed design.

**Table 5:** Comparison of the conventional WRSM with the proposed BLWRSM.

Parameter	Units	CWRSM	Proposed BLWRSM
SW current (per phase)	A_rms_	2.227	2.227
Torque (average value)	Nm	8.963	8.644
Torque ripples	%	0.78	0.694
FW current	A (average value)	1.32	1.323
HW current	A_rms_	0	1.3232
Core loss	W	54.37	56
Copper loss in SW	W	35.71	35.71
Copper loss in HW	W	0	0.56
Copper loss in FW	W	3.903	3.921
Efficiency	%	89.988	89.44

Although the proposed machine exhibits a slight efficiency reduction of 0.61%, the BLWRSM configuration removes the need for any external DC source, slip rings, and brushes, thereby improving operational reliability, reducing maintenance requirements, and avoiding mechanical wear components and sparking, making it suitable for fire-hazardous environments. Therefore, the results confirm that the proposed BL topology maintains comparable electromagnetic performance while providing the practical advantages of BL operation.

### 3.4. Comparative analysis of the existing BL design and the proposed BL design

The proposed BL motor design and the existing BL motor design [26] were also compared to assess relative performance. Ensuring fair comparison, the two motors were designed with identical stator and rotor inner and outer dimensions, and the same air-gap length. The SW turns/phase, the FW and HW turn counts, the number of FW poles, and the rotational speed were all kept identical for both machines.

After analysis, it is observed that the proposed BLWRSM topology offers advantages over the existing BLWRSM. For the existing BL machine, the average measured torque is 8.24 Nm, while that of the proposed BL design is 8.644 Nm. This indicates that the proposed motor achieves about a 4.90% increase in average output torque compared to the existing BL design [26]. Similarly, the torque ripple in the proposed BLWRSM is 0.694%, whereas the existing BLWRSM exhibits 18.41% torque ripple [26]. This reveals that the proposed BLWRSM achieves approximately a 96% reduction in torque ripple compared to the existing design. [26].

The SW input current provided to the proposed machine is 2.227 $A_{\text{rms}}$ at 60 Hz, whereas the input current for the existing BL-WRSM [26] is 4.5 $A_{\text{rms}}$ at the same frequency. In comparison to the existing BL design [26], the proposed BL configuration delivers 4.85% higher output power by drawing 50.5% lower input current. The reduced stator current also results in lower copper losses relative to the conventional BL machine [26] and improves the overall efficiency of the proposed motor as well. A detailed comparison between the two machines is given in Table 6. The results show that the BLWRSM, proposed in this research work, achieves higher output power with reduced torque ripples compared to the existing BL machine [26].

**Table 6:** Comparative performance assessment of the proposed and existing BLWRSM [26].

Parameters	Units	Existing BLWRSM [26]	Proposed BLWRSM	% Improvement in proposed BLWRSM
Output power	W	777	814.6778	4.85
Torque	Nm	8.24	8.644	4.90
Torque ripples	%	18.41	0.694	−96.23
SW current (per phase)	A_rms_	4.5	2.227	−50.51

**Fig 21:**
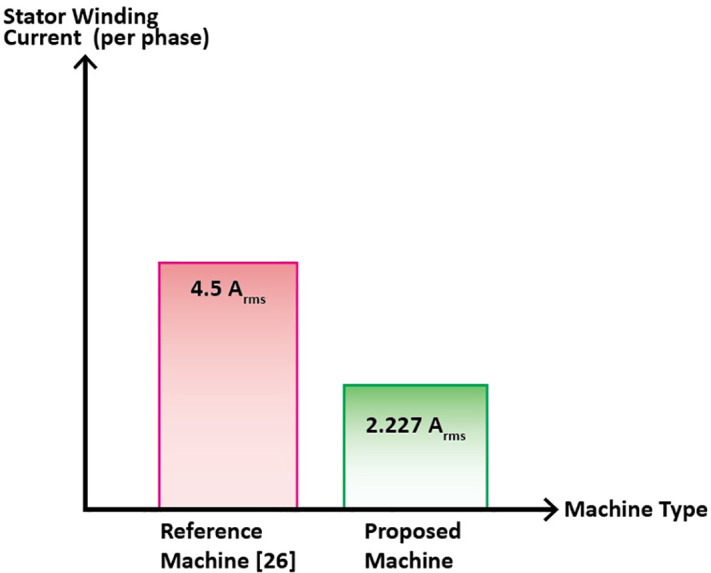
Comparison of stator winding currents used in reference and proposed machines.

**Fig 22:**
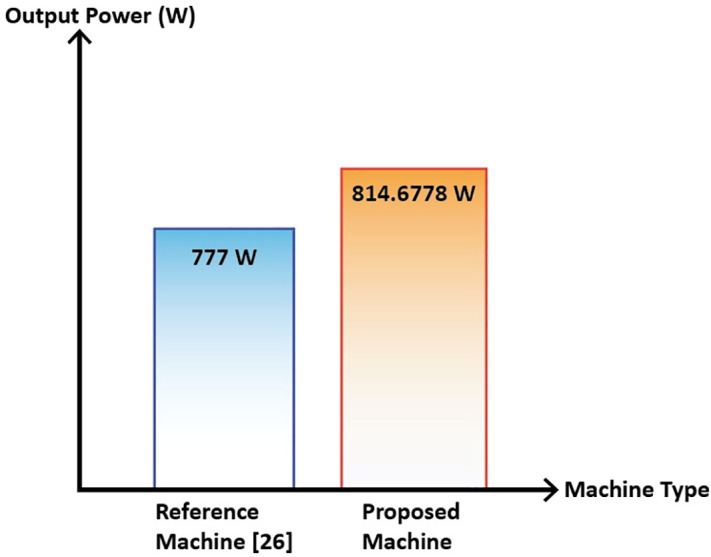
Comparison of output power for reference and proposed machines.

**Fig 23:**
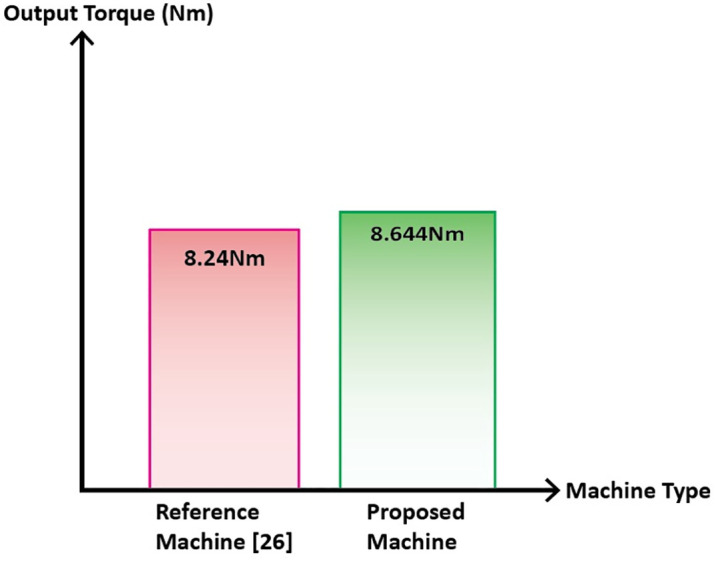
Comparison of output torque for reference and proposed machines.

**Fig 24:**
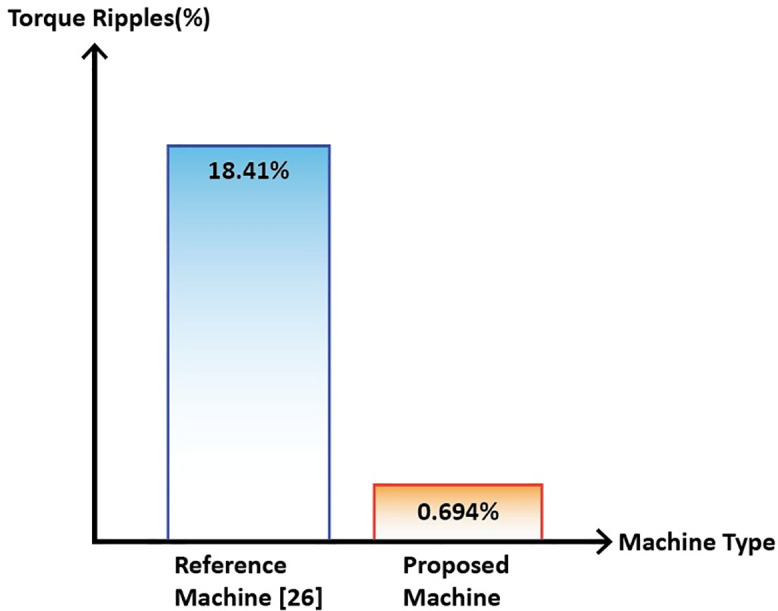
Torque ripple comparison between the reference and proposed machines.

As shown in Figs 21–24, the proposed BLWRSM demonstrates superior performance compared to the reference model. It requires 50.5% less SW current, delivers 814.68 W output power—4.86% higher than the reference machine’s 777 W—and produces 8.644 Nm torque, a 4.90% increase over the reference machine. Furthermore, the torque ripples are significantly lower at 0.7%, compared to 18.4% in reference design, highlighting the enhanced efficiency and overall performance of the proposed configuration.

Torque ripples, efficiency, average output torque, etc., are significant parameters to assess the performance of BLWRSM topologies. A comparative summary of these parameters of the proposed design presented in this work with the state-of-the-art BLWRSM topologies is given in Table 7.

**Table 7:** Performance comparison with state-of-the-art BLWRSM topologies.

References	Year	Topology	Torque ripples (%)	Average output torque	Efficiency
[27]	2016	Topology employing two inverters and permanent magnets.	18.5%	4.95 Nm	75.32%
[28]	2020	Topology is based on a single inverter with two extra diodes to generate fundamental and third-harmonic MMF.	34.05%	5.2724 Nm	Not specified.
[24]	2020	Stator HW based BLWRSM topology	13%	4.5 Nm	83%
[16]	2020	Hysteresis controller-based topology to generate third-harmonic in the sMMF.	40.46%	8.143 Nm	92.99%
[29]	2021	Third-harmonic component based BLWRSM topology.	47.06%	1.661 Nm	Not specified.
[30]	2022	Two SWs-based topology powered by two inverters.	75%	7.15 Nm	77.15%
[31]	2022	Hysteresis controller-based topology for producing third-harmonic in the sMMF.	34.88%	6.88 Nm	Not specified.
[22]	2023	BLWRSM topology using subharmonic excitation	16.10%	5.55 Nm	87.81
[23]	2023	Topology based on outer-rotor structure employing SHC.	39.77%	3.03 Nm (in spin mode)	84.97
[32]	2023	Wound rotor vernier motor-based topology with additional single-phase auxiliary winding	22.48%	3.85 Nm in spin mode	66.24%
[33]	2023	SHC based topology with two stators.	15.18	8.5 Nm	80.3%
[34]	2025	Third-harmonic component based BLWRSM topology.	80%	6 Nm	71.2%
Proposed topology	2026	Topology with special SW scheme employing single inverter to generate a SHC.	0.694%	8.644 Nm	89.44%

## 4. Conclusion

This paper introduces a novel brushless wound rotor synchronous machine (BLWRSM) configuration with a unique stator winding (SW) scheme. Two series-connected sets of unequal turns, forming the unique SW scheme of the proposed machine, generate an additional subharmonic component (SHC) in the stator magnetomotive force (sMMF). The sMMF is predominantly composed of SHC and the fundamental component (FC). The SHC assists in achieving the brushless (BL) operation of the proposed configuration while the main magnetic field within the air gap is produced by the FC. The SHC and rotor harmonic winding (HW) have an equal number of poles. Alternating current (AC) is developed in the HW by the airgap sMMF’s SHC and is then rectified and supplied as direct current (DC) to the rotor field winding (FW) to achieve BL excitation. The proposed machine’s rotor configuration integrates HW, a bridge rectifier, and FW. The proposed configuration is validated by performing 2-Dimensional finite element analysis on JMAG software for an 8-pole/12 slot machine. The results demonstrate that the machine achieves more torque at rated speed with less torque ripples. Due to its BL nature, the proposed configuration eliminates the requirement for slip rings and carbon brushes, which are the main limitations of the conventional wound rotor synchronous machines. Similarly, the proposed configuration requires a single inverter, resulting in a reduction of system size and cost by eliminating the need for a dual inverter. Additionally, in comparison to the existing BL configuration [26], the proposed configuration delivers improved average torque while requiring less SW current, making it ideal for high-torque applications.
